# Expanding the reach of evidence-based mental health interventions to private practice: Qualitative assessment using a policy ecology framework

**DOI:** 10.3389/frhs.2022.892294

**Published:** 2022-07-22

**Authors:** Hannah E. Frank, Lauren Milgram, Jennifer B. Freeman, Kristen G. Benito

**Affiliations:** ^1^Warren Alpert Medical School of Brown University, Providence, RI, United States; ^2^Bradley Hospital, East Providence, RI, United States

**Keywords:** policy, mental health, implementation, determinants, private practice

## Abstract

**Background:**

Evidence-based interventions (EBIs) for mental health disorders are underutilized in routine clinical practice. Exposure therapy for anxiety disorders is one particularly difficult-to-implement EBI that has robust empirical support. Previous research has examined EBI implementation determinants in publicly funded mental health settings, but few studies have examined EBI implementation determinants in private practice settings. Private practice clinicians likely face unique barriers to implementation, including setting-specific contextual barriers to EBI use. The policy ecology framework considers broad systemic determinants, including organizational, regulatory, social, and political contexts, which are likely relevant to EBI implementation in private practice settings but have not been examined in prior research.

**Methods:**

Qualitative interviews were conducted to assess private practice clinicians' perceptions of EBI implementation determinants using the policy ecology framework. Clinicians were asked about implementing mental health EBIs broadly and exposure therapy specifically. Mixed methods analyses compared responses from clinicians working in solo vs. group private practice and clinicians who reported high vs. low organizational support for exposure therapy.

**Results:**

Responses highlight several barriers and facilitators to EBI implementation in private practice. Examples include determinants related to organizational support (e.g., colleagues using EBIs), payer restrictions (e.g., lack of reimbursement for longer sessions), fiscal incentives (e.g., payment for attending training), and consumer demand for EBIs. There were notable differences in barriers faced by clinicians who work in group private practices compared to those working in solo practices. Solo private practice clinicians described ways in which their practice setting limits their degree of colleague support (e.g., for consultation or exposure therapy planning), while also allowing for flexibility (e.g., in their schedules and practice location) that may not be available to clinicians in group practice.

**Conclusions:**

Using the policy ecology framework provides a broad understanding of contextual factors that impact private practice clinicians' use of EBIs, including exposure therapy. Findings point to potential implementation strategies that may address barriers that are unique to clinicians working in private practice.

## Introduction

Evidence-based interventions (EBIs) are infrequently used in routine clinical care settings. Prior research has largely focused on strategies to improve the implementation of EBIs in publicly-funded mental health settings. However, less is known about the implementation of EBIs in private practice mental health settings, where there is also a research-practice gap. Private practice settings represent a large sector of the mental health workforce (1), including a plurality (44.8%) of psychologists (2), and serve a large portion of people with private insurance. Treatment access disparities are particularly wide among individuals with public insurance, but privately-insured individuals also face significant barriers to accessing care (3, 4). Estimates indicate that ~40% of youth with private insurance do not receive needed mental health services (5, 6) and that these families face significant barriers to receiving mental health care (7, 8). The number of unmet mental health needs, especially for anxiety and depression, has only been exacerbated as a result of the COVID-19 pandemic (9). Thus, identifying strategies to increase EBI use in private practice settings may improve access to care for a large portion of individuals in need of mental health services.

Existing research in private practice settings provides some evidence that there are unique challenges to EBI implementation that may be specific to this setting. For instance, in one study, private practice clinicians were found to hold more negative global attitudes toward EBIs than those working in public outpatient settings (10). One interpretation the authors provide for this finding is that private practice clinicians may have chosen this setting to allow them more autonomy and fewer mandates related to the types of interventions they are expected to deliver. Another study, focused on evidence-based assessment, found lower rates of evidence-based assessment use among private practice clinicians compared to clinicians in other settings (11). Supervisory practices reflect these general trends, with fewer references to EBIs and less supervision time spent discussing them among clinicians working in private practice settings (12). Prior studies have not specifically examined determinants of EBI use in private practice settings, which is a necessary first step to inform future work focused on supporting EBI implementation in this setting.

One intervention that is particularly underutilized is exposure therapy for anxiety, which has strong evidence for its efficacy but is rarely used in practice settings (13). A study of private practice clinicians working in Germany found that issues related to the practicability of exposure (e.g., feasibility of conducting exposures in session), negative beliefs about exposure, and distress for the therapist in delivering exposure therapy were barriers to its delivery (14). These findings are consistent with previous research indicating that therapists' negative beliefs about exposure are a primary barrier to its delivery (15–17). Although some interventions have been developed to directly address these negative beliefs (18), insufficient access to training in exposure is another commonly endorsed barrier (19). Even when therapists do receive training, actual use of exposure remains somewhat limited (20). Receiving consultation after training is one promising method that has been shown to increase use of exposure-based treatments (21). However, therapists in private practice have been found to use sub-optimal exposure techniques, such as assigning client self-directed exposure rather than conducting exposures in session (22). This may be due to difficulty accessing ongoing consultation, or a result of various other factors such as competing demands and limited organizational support.

Existing research on determinants of EBI use more broadly have identified an array of clinician- and organization-level barriers that interfere with implementation (23–26). For instance, organizational factors, such as proficient culture, leadership, and presence of champions influence the implementation of EBIs (27). Clinician-level barriers have also been identified as predicting EBI implementation, such as competing responsibilities and lack of training (28, 29). Even when clinicians do access training, one-time or even intensive trainings are not sufficient to lead to sustainable behavior change (30–32). In addition to these clinician- and organization-level considerations, there are also even broader contextual determinants that influence EBI implementation. In their “policy ecology” framework, Raghavan et al. (33) highlight the importance of considering multiple levels of the ecology of implementation, ranging from the organizational context to the larger social context in which implementation takes place, to ensure successful implementation of EBIs. This framework incorporates factors such as incentive strategies for policymakers and payers to improve EBI implementation. According to the policy ecology framework, efforts to sustainably implement EBIs will require implementation strategies that expand beyond clinical factors and include a focus on systemic or ecological determinants. Such determinants have been increasingly studied in public sectors [e.g. (34)], but have not been specifically examined in private practice settings.

**Figure 1:**
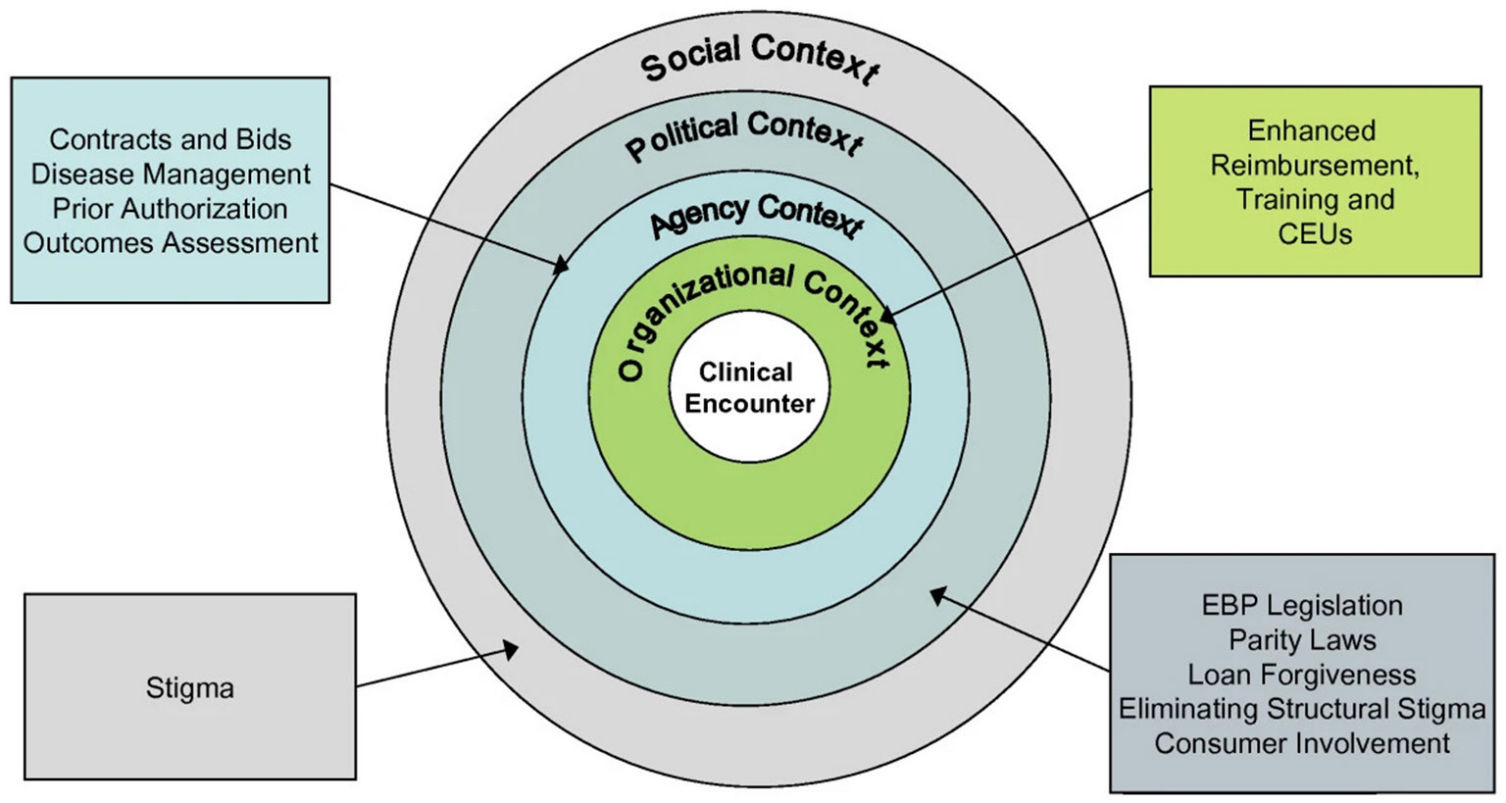
A policy ecology of implementation. Reproduced from (33); distributed under the creative commons attribution license.

The policy ecology framework highlights four key levels that influence implementation, including the organizational context, the agency (regulatory) context, the social context, and the political context, as shown in Figure 1. The organizational context refers to the mental health practice in which treatment is delivered, which for private practitioners may consist of one individual or a large group practice. Specific considerations within organizations include: (1) the costs of delivery that organizations accrue (e.g., for ongoing supervision), which are typically not reimbursed, as well as (2) continuing education, which is often provided through or subsidized by organizations. Policy levers at the organizational level may include adjusting state licensing boards' requirements and expectations regarding continuing education to emphasize EBIs. However, therapists who work in private practice may face financial barriers to accessing continuing education and place less of an emphasis at the organizational level on implementing new EBIs (35). For instance, in an assessment of psychologists' use of outcome measures (36), clinicians in private practice settings were more likely to use outcome measures for clinical purposes, whereas clinicians in other settings (e.g., schools, community mental health, outpatient clinics) endorsed using these measures for clinical *and* business reasons (i.e., requirements by the work setting). Understanding how contexts for private practice clinicians may differ from other settings can inform the tailoring of implementation strategies.

The second level of the policy ecology is the agency (regulatory) context, which refers to payers (i.e., insurance companies) or states. Specific considerations within this level include: (1) fiscal incentives, such as pay-for-performance and public recognition for providing EBIs; and (2) payer restrictions, such as requirements for prior authorization or limits to the number and frequency of therapy sessions. In contrast with clinicians who work in publicly-funded agencies, private practice clinicians in the United States are not required to accept insurance. This is likely to have implications for how private practice clinicians interface with payers, including having more flexibility with the decision about whether to panel with insurance companies. If payers are to have an influence on the practice of private practitioners, they will likely need to incentivize providers to accept health insurance plans *via* strategies such as higher reimbursement rates, coverage of more sessions, and reduced administrative burden.

The political context level refers to legislative and advocacy efforts that may affect EBI use. From a consumer perspective, this may include efforts related to improving accessibility and affordability of mental health treatment for consumers. In public agencies, research has examined how policy mandates related to EBI use influence clinicians' behavior. In general, policy makers are urged to avoid strict policy mandates and instead consider how to balance EBI expectations with available support from a system (33, 34). One study demonstrated modest gains in cognitive behavioral therapy use following a system-wide initiative focused on supporting EBI implementation, a finding that was moderated by organizational culture (37). Given that many policy mandates may be less applicable to private practice clinicians, especially those who do not accept insurance, other issues at the political level may have more relevance. For instance, EBI training is not consistently required during pre-service training for doctoral and master's level clinicians (38). Existing accreditation standards for graduate programs may make it challenging to prioritize EBI training (39, 40). Thus, efforts focused on changing accreditation standards and increasing the emphasis on EBI-based training in pre-service settings may be one relevant lever at the political level that could influence private practice clinicians' behavior (39).

Finally, the social context level refers to public perceptions of EBIs, especially related to stigma and discrimination, as well as consumer demand for EBIs. A systematic review of parents' perceptions of barriers and facilitators to seeking mental health treatment demonstrated that parents frequently reported a lack of knowledge about where or how to seek treatment (41). Direct-to-consumer marketing is one approach that has been proposed to address stigma and increase consumer demand for EBIs (42), which in turn may motivate private practice clinicians to provide them.

Although the levels of the policy ecology framework were originally developed with public mental health settings in mind, the present study assesses how each of its levels might also apply to private practice mental health settings. To date, incentive strategies beyond providing training and technical assistance are rarely used (43) and limited research has examined the barriers to EBI adoption in private practice settings. The present study uses the policy ecology framework (33) to assess private practice therapists' perceptions of multi-level contextual factors that influence their use of EBIs broadly, and exposure therapy specifically. Exposure therapy was selected as a specific example of an EBI given that it has been one of the most difficult to implement (13, 22). Although an array of intervention-specific clinician- and organization-level barriers to exposure implementation have been identified, there may also be opportunities to engage implementation strategies at a broader ecological level to increase adoption and sustained use of exposure therapy. The primary aim of the present study was to conduct qualitative interviews with private practice mental health clinicians to identify incentive structures that may affect EBI implementation, with a particular emphasis on exposure therapy for anxiety disorders. Mixed methods analyses were used to examine differences between therapists in solo vs. group private practice settings, given that each practice structure is likely to present unique implementation considerations. In addition, we examined whether qualitative responses differed based on therapists' perceived level of organizational support for implementing exposure therapy.

## Method

### Participants

Participants include therapists (*N* = 20) with (1) an advanced degree in a mental health field who (2) work in private practice settings. Given that exposure therapy is a particularly difficult-to-implement EBI, we were interested in understanding responses from clinicians who had and had not sought out explicit training in exposure therapy. Thus, purposive sampling was used to identify approximately equal numbers of participants with and without previous training in exposure therapy. The final sample included *n* = 9 (45%) therapists who had previously participated in full day or longer training focused on exposure therapy and *n* = 11 (55%) who had never attended an exposure-focused training.

### Measures

De-identified survey data were collected and stored using Research Electronic Data Capture (REDCap), a HIPAA compliant web-based survey platform (44, 45).

#### Demographics form

A demographics questionnaire assessed participants' age, gender, race, and education. It also assessed topics related to the participants' work setting, theoretical orientation, and level of familiarity with exposure therapy.

#### Organizational innovation-specific capacity for exposure (OISCE)

The OISCE (23) assesses therapists' perceptions of organizational policies and procedures for supporting exposure use in their clinical setting with responses rated from 0 (not at all) to 4 (extremely). The measure includes 17 items that cut across five domains of interest, including supervisory support, collaboration, organizational policies, resources, and emphasis on exposure.

#### Qualitative interviews

A semi-structured qualitative interview guide (see Supplementary Material) included prompts about participants' experience using EBIs in their practice, as well as their training and consultation needs for EBIs. Given that exposure therapy is a particularly underused EBI with strong evidence for its efficacy, therapists were also specifically asked about their use of and training in exposure therapy. In addition, participants were asked about barriers and facilitators to using exposure and other EBIs. Questions were developed based on the policy ecology framework (33), which includes factors at the organizational, agency, political, and social context levels.

### Procedure

All study procedures were approved by the Lifespan Institutional Review Board (IRB). Participants with previous training were recruited by contacting therapists who completed prior studies at the Pediatric Anxiety Research Center (PARC) and agreed to be contacted for future research. Additional participants with and without previous training were recruited by sending emails to local (New England) professional listservs and contacting private practice clinicians on referral lists maintained at Bradley Hospital. Local private practice agencies who had publicly available contact information were also emailed and asked to distribute information about the survey to providers in their practice. Finally, participants who completed the qualitative interview were asked if they were willing to forward information about the study to their colleagues as part of a “snowball” recruitment method.

Recruitment of participants was informed by the Dillman Tailored Design Survey Method (46, 47). Initial contact included a phone call or email to potential participants, as well as two follow-up emails sent 1 week after the initial contact requesting participation in the study. Participants who indicated interest in the study were emailed a REDCap link to complete online quantitative measures prior to qualitative interviews. Then, participants were contacted to complete the qualitative interview. Our recruitment methods do not allow us to estimate how many people may have received information about the study. However, a total of 39 people initiated the survey questionnaires, and 24 of them completed the survey measures and provided their contact information. Four of those participants were either not available or did not respond to attempts to schedule the qualitative interview. All 20 participants who were scheduled for qualitative interviews completed them as scheduled. Interviews were audio recorded and conducted one-on-one with participants by phone until thematic saturation (i.e., no emergence of new concepts) (48) was reached. Completion of qualitative and quantitative measures took a total of ~60–90 min. There was no additional follow-up with participants after completion of these interviews. Interviews were conducted by a female postdoctoral fellow (HEF) and a female advanced doctoral student who did not have previous relationships with the study participants. Participants were told that interviewers were affiliated with PARC and interested in understanding factors that affect clinicians' use of evidence-based interventions, including exposure therapy. The first author (HEF) has previous experience leading qualitative studies and provided training to the graduate student to conduct interviews. Interviews were transcribed using NVivo transcription services, checked by undergraduate research assistants, and spot checked by the first author (HEF).

### Qualitative coding and data analysis

Qualitative interviews were analyzed in NVivo using content analysis (49) informed by Raghavan and colleagues' (33) policy ecology Framework. A priori codes included: (1) organizational context and its two subcodes: costs of delivery and continuing education; (2) agency (regulatory) context and its two subcodes: fiscal incentives and payer restrictions; (3) social context; and (4) political climate. Coders included the first author (HEF) and a research assistant (LM) who received training in qualitative analysis from the first author. The coders collaboratively reviewed six transcripts to inform their iterative development of a codebook. They then independently applied codes to two transcripts to determine initial interrater reliability (*kappa* = 0.64). Finally, both coders independently coded transcripts with 20% overlap (*n* = 4 transcripts) to assess final reliability (*kappa* = 0.82). At each stage of coding, disagreements were resolved through discussion and consensus.

Thematic analysis of codes was conducted in Excel by the same authors who completed coding of transcripts (HEF and LM). Codes were examined collaboratively to identify patterns and key themes through discussion. Mixed methods analyses were used to integrate quantitative and qualitative findings using a convergent design in which quantitative and qualitative data were merged (50). Quantitative data (i.e., solo vs. group practice; high vs. low organizational support for exposure based on a median split on the OISCE) was entered into NVivo as attributes of each participant and used to categorize and compare themes among subgroups. Once exported into Excel, content analysis was used to identify themes for each code. Then, coders collaboratively created brief written summaries for each theme and for each quantitative variable (i.e., solo private practice, group private practice, high organizational support, low organizational support). These summaries were compared to identify differences in qualitative responses for each quantitative variable. All qualitative analyses adhered to consolidated criteria for reporting qualitative research (COREQ) (51).

## Results

Participants were predominantly female (*n* = 18, 90%) and white (*n* = 18, 90%). The majority had doctorate degrees (*n* = 13, 65%) and worked in a solo private practice (*n* = 11, 55%). Scores on the OISCE indicated overall low levels of organizational support for exposure therapy (*M* = 1.71, *SD* = 0.93). Clinicians with high organizational support for exposure had scores above the median of 1.85, indicating responses of “somewhat” or above. See Table 1 for additional demographic information. Table 2 includes an overview of study codes, themes, and example quotes, as well as suggested implementation strategies to address barriers related to each theme.

**Table 1:** Participant demographic characteristics (*N* = 20).

**Variable**	***M*** **(*****SD*****) or** ***N*** **(%)**
Age	46.25 (13.86)
**Gender**	
Female	18 (90%)
Male	2 (10%)
**Race**	
More than one race	1 (5%)
Southeast Asian	1 (5%)
White	18 (90%)
**Ethnicity**	
Hispanic or latine	0 (0%)
Not hispanic or latine	20 (100%)
**Degree**	
Doctorate	13 (65%)
Master's	7 (35%)
**Exposure training**	
Yes	9 (45%)
No	11 (55%)
**Practice type**	
Solo	11 (55%)
Group	9 (45%)
**Professional discipline**	
Clinical psychology	13 (65%)
Social work	3 (15%)
Marriage and family therapy	1 (5%)
Counseling	3 (15%)
**Theoretical orientation^a^**	
Cognitive behavioral	8 (40%)
Eclectic	4 (20%)
Feminist	3 (15%)
Family systems	4 (20%)
Humanistic	1 (5%)
Solution-Focused	1 (5%)
Strengths-Based	1 (5%)
Third wave	2 (10%)
Not reported	5 (25%)
**Populations treated^a^**	
Adults	18 (90%)
Children	13 (65%)
Couples	7 (35%)
Families	9 (45%)

a Participants could endorse more than one option; thus, totals are >100%.

**Table 2:** Reported barriers and facilitators and potential implementation strategies.

**Code**	**Theme**	**Quotes**	**Implementation Strategy**
Organizational context	Organizational policies that support or impede EBI use	“I think some of our higher-ups [...] they don't want us to depend on going out into the community if we don't have to” (P2037)	Adopt organizational policies that align with EBI implementation processes
	Supervisor support for or knowledge of EBIs	“I can imagine that in a group practice, unless it's an OCD group practice, I think people would have a hard time with [exposures that involved things like] smoke bombs, knives, gagging, sticking yourself with a straight pin that has been sterilized” (P8010)	Offer EBI training that is tailored specifically for supervisors
	Colleague support for or knowledge of EBIs	“When I had to do an exposure or when I've been asked to be part of an exposure, people in the office are really willing and able to be a part of that. That's obviously super helpful. Front office staff will even get involved” (P1616)	Support the development of peer consultation groups
Continuing education	Availability of consultation	“What I pick up in the workshop is the extent of what I learn. Or similarly, I went to a national training—a two day ACT training—and it was super interesting. But again, I have no ongoing supervision or education beyond my peer consultation groups” (P1613)	Provide supervisor consultation and/or facilitate peer consultation
		“When I was a postdoc, we had formal group supervision, which I loved. And then when I became a psychologist… you don't do it anymore” (P1616)	Encourage licensing board to allow for receiving consultation to count toward required CEUs
	Access to online resources	“Google Scholar can be overwhelming and unhelpful… especially if you don't have access to all the journal articles. If you're not in like a university setting”	Develop and distribute routinely updated educational materials such as online toolkits
	Compensation for training	“The hard thing with training, like you're both not getting paid for the time and you're paying [to receive the training]” (P3004)	Compensate clinicians for lost billable hours to attend training in EBIs (potentially with free CEUs)
Cost of delivery	Compensation for collateral contact and preparation time	“I feel like from a clinical perspective, there's not much incentive for clinicians to do exposure exercises outside the office. Which can be pretty limiting” (P1226) “… We're like teachers and we prep and we have worksheets. Every client, I have to remember where I am in the protocol and what's next?” (8010)	Create a Decision flowchart to help prioritize session preparation and collateral contact
	Availability of supplies and exposure stimuli	“If you need the client to meet you at Wal-Mart [for an exposure], then do they need a cab voucher or is that something we can provide for them?: Do we need to set up people who are gonna ask them questions [for exposures]?” (P4025)	Allot funds for buying supplies Provide list of key supplies for exposures/EBIs
Agency (regulatory) context			
Payer restrictions	Ability to bill for longer and/or more frequent sessions	“I only take [Insurance Company name]. I used to take a wider range of insurances. [Now,] I take [only one Insurance Company] and I do sliding scale. I don't like people telling me what I can do” (P1526)	Create billable codes that permit longer and/or more frequent sessions for EBIs that have documented evidence of their benefits
		“A lot of the insurances... you really have to fight for more than a 45 minute session… So I just do 55 minute sessions anyway… So I just don't get paid for those” (P2804)	
	Payer knowledge about EBIs	“I can [bill for a longer session], but I know that I'm going to have an hour of my time wasted for a care management call with a person who doesn't even know what I'm talking about when I list the evidence-based treatments” (P8010)	Provide education to care managers about EBIs
	Cost to families	“If there was some sort of parent session that was supposed to coincide with the parent session, they would be like “oh can we just do it right after?” I [would say], “Yeah, but insurance isn't going to pay for that.” So, then what? That's not fair to the family when time wise that would be the most feasible” (P2037)	Reduce cost of families Reduce discrepancies in reimbursement rates across different insurance companies and plans Increase transparency about reimbursement rates
Fiscal incentives	Training incentives	“Once you're a licensed clinician and you make a decent amount of money, it's so much easier to bill people than to do things where you're not getting paid. Or you'll have to pay money” (P1226)	Insurance companies recognize and label providers who are certified or have specialized training in EBIs
	Reimbursement for EBI use	“Well, I think everyone wants to be paid more, but my feeling is that if you're practicing, a licensed practitioner, you need to be using evidence-based practices” (P4610) “Oh, well, paying me more would be really motivating. Yes. I mean, if I could even if I could easily get paid for like the extra planning time or anything like that. That would be great” (P2804)	Insurance companies reimburse training in EBIs Provide enhanced rate of compensation for EBI use
Political context	None	N/A	N/A
Social context	Consumer education in EBIs	“Yeah, I know it's hard because a lot of clients don't know necessarily to come in and ask for exposure” (P3004)	Partner with community organizations and providers (e.g., primary care physicians) to provide education that meets communities' mental health education needs
		“I would guess-timate 15–20% [of clients] may know about evidence-based practice” (P4610)	Partner with patient advocates to provide education about EBIs
	Consumer demand for EBIs	“Some people come in and [exposure] is what they want. So that makes it easy” (P1616)	Use tailored marketing strategies to promote EBIs directly to consumers

### Organizational context

Clinicians described varying levels of organizational support for EBI use. Among private practice clinicians, the “organizational context” refers to the individual practitioner, as well as the setting (e.g., geographical location, office space, proximity to colleagues) in which they work. Organizations that placed more emphasis on EBI use were described as facilitating EBI implementation. Specifically, organizations that offered EBI-consistent in-house training (e.g., through case conferences) fostered a “push for evidence-based interventions” (P1226). Many clinicians described working in organizations that support the implementation of cognitive behavioral therapy (CBT) principles while also emphasizing that “the patient's needs come first” (P1004) and that CBT should be applied flexibly. Some clinicians described their organizations as supporting EBI use conceptually, but not supporting specific aspects of EBIs (e.g., inability to hold sessions in the community or travel with clients during sessions). Supervisors were generally described as being supportive of EBI use. Similarly, clinicians endorsed that the presence of colleagues who also use EBIs can support EBI implementation.

Clinicians identified some organizational contextual factors that were specific to implementing exposure therapy. For instance, clinicians mentioned that having colleagues who understand and can help with exposure can facilitate exposure implementation. One clinician described, “when I had to do an exposure or when I've been asked to be part of an exposure, people in the office are really willing and able to be a part of that” (P1616). Clinicians also reported that space constraints affect their ability to conduct exposure work in the office. One clinician described, “I think that exposure therapy is best done if the clinician is able to go out with the person and there's more intensive therapy or if it's a home-based program. I don't feel like in private practice I would feel as comfortable” (P2605). Clinicians described that having businesses nearby that are willing to assist with exposures can facilitate the completion of exposure work outside of the office. Finally, clinicians noted that it is helpful to have knowledge of local providers who use exposure if they need to refer to a specialist, but that referral options are often limited [e.g., “There is nobody in (geographic region) who does hard core exposure stuff” (P1058)].

#### Mixed methods analyses

There were notable differences in descriptions of organizational support for EBI use between clinicians in solo vs. group practice. As might be expected, clinicians working in group practices more often reported that supervisor and colleague support are available to them, whereas clinicians working in solo practice hardly mentioned supervisor support for EBI use and varied more in their reports of the availability of colleagues who support EBI use. Clinicians in group practice more often mentioned that being busy (e.g., scheduling back-to-back sessions) makes it difficult to leave the office for exposure. However, as one clinician stated, “having a lot more people around, in my opinion, makes it easier to do exposure” (P2621). Clinicians working in solo practice more often described leaving the office to meet with clients, although they still cited some barriers and restrictions. One clinician in solo practice mentioned that not having other clinicians in the office is a barrier to the completion of social exposures. Although they navigate around this by identifying social exposures that can be completed with just the clinician and the patient, they said, “it would be great to have other people, unfamiliar people, that [the patient] could interact with” (P4610).

There were also differences in descriptions of organizational support for EBI use for clinicians with high and low organizational support. Clinicians with high organizational support provided examples of the ways in which their organization supported EBI use, including providing “in-house” continuing education and consultation on EBIs, as described in more detail below. Few such examples were given by clinicians with low organizational support. Clinicians with high organizational support noted the importance of supervisors for support and education in EBIs, whereas clinicians with low organizational support did not often discuss the role of supervisor support in EBI implementation. Clinicians with high organizational support spoke about how their colleagues give “a lot of feedback that is often guided by research or something people have done in their own practice” (P1226). They also described having colleagues to help with in-office exposures. In contrast, clinicians with low organizational support spoke about their setting feeling like “you're just here on an island” (P2605).

#### Continuing education

Clinicians described that the availability of organization-sponsored training (e.g., refresher courses on certain topics) would be helpful to facilitate EBI implementation. Clinicians described that having training “in-house” would facilitate training attendance, and that attending training with colleagues would facilitate group discussion after training. On the other hand, clinicians noted that informal training through case discussions within their practice may not be as helpful as formal trainings. One clinician described, “I would probably [want] a little more formal than what my practice is doing now in terms of training. I don't mind case conferences, but honestly, I don't always go” (P2621). The same clinician described that informal training is “helpful to some degree,” (P2621) but may be less effective if the group is too large or more focused on “brainstorming” (P2621) rather than concrete suggestions. Clinicians indicated that compensation for attending training would facilitate training attendance and subsequent EBI implementation; they described that the cost of getting training includes both the actual cost of the workshop, as well as the lost income from not seeing patients during that time. Clinicians also said that receiving compensation in the form of continuing education units (CEUs) would facilitate training attendance. Clinicians highlighted the value of consultation to support ongoing EBI implementation after training. They described that it is easy to forget training content over time, and that isolated training workshops are often not sufficient to sustain EBI implementation without ongoing consultation. Clinicians indicated that both peer and expert consultation can be helpful, and that it is valuable to hear a diversity of perspectives through consultation.

Clinicians cited various sources through which they receive training. One common example was state-wide, discipline-specific organizations such as the Rhode Island Psychological Association as well as national organizations such as Anxiety and Depression Association of America (ADAA). Clinicians reported that training resources from professional organizations can be helpful, although the cost can be a barrier. Clinicians described online resources as a lower-cost (or no-cost) way of seeking training and continuing education, including professional mailing lists, listservs, special interest groups, Google Scholar, and Facebook groups. These resources were described as helpful to support EBI implementation, particularly when a clinician has a question about a specific clinical topic. Lastly, clinicians noted that being affiliated with an academic institution (e.g., as an adjunct professor) provides access to additional resources such as journal articles, training, and grand rounds that can facilitate continuing education and EBI implementation.

##### Mixed methods analyses

There were some differences in descriptions of continuing education between clinicians in solo vs. group practice. On one hand, clinicians in group practice reported that the presence of other clinicians made it more likely that they could find someone who could provide consultation. On the other hand, scheduling conflicts and busy schedules (“we joke we're passing ships in the night;” P6303) were described as practical barriers to receiving consultation from colleagues in group practice. Clinicians in solo practice cited specific professional organizations they have joined and specific conferences they have attended. For instance, one solo practice clinician described, “I joined… ADAA and I just started being very alert to opportunities” (P1058). Clinicians in both groups described being aware of training resources, but those in solo practice described more actively using resources outside of their organization. In terms of differences by level of organizational support, clinicians with high organizational support reported finding consultation that was helpful—even if it was outside of their organization [“In [name of professional group], we have a good amount of people you can always consult with” (P8907)]. In contrast, clinicians with low organizational support reported more mixed success in finding helpful consultation, as indicated by statements such as, “there are definitely times when I go, ‘hmm I wish there was someone I could run this by”' (P4610).

#### Costs of delivery

In addition to the financial considerations related to payer restrictions, clinicians cited costs associated with EBI delivery. Clinicians described that collateral contact with clients, insurance companies, other providers, and hospitals can occupy a lot of time that clinicians are spending unpaid. Clinicians mentioned that they try to avoid collateral contact when possible (e.g., by not having an office phone), but that some collateral contact is unavoidable. Clinicians also highlighted that preparing before a session requires time and effort that is unpaid. Clinicians cited supplies (e.g., rewards for children) as a cost that they incur without reimbursement, and that having supplies available and/or reimbursement for supplies would be helpful. Clinicians specifically described that conducting exposure in session requires additional preparation time, session time, materials, and ability to leave the office. One clinician said, “In order to use [exposure], there's a ton of prep work that goes into it” (P2605).

##### Mixed methods analyses

There were few differences in descriptions of cost of delivery between clinicians working in solo vs. group practice. Clinicians working in solo practice and clinicians with low organizational support mentioned wanting reimbursement for preparation time, as indicated with comments such as, “Money for planning time would change my behavior in that I would probably [treat] more children… because they're harder to plan for” (P2804). In contrast, clinicians in group practice or with high organizational support rarely mentioned this. Similarly, clinicians with low organizational support mentioned the cost of purchasing supplies [“I end up going out and buying things for kids (like) toys and prizes and all that” (P2804)], whereas no clinicians with high organizational support mentioned this.

### Agency (regulatory) context

#### Payer restrictions

Clinicians described that payers generally want providers to use EBIs, particularly CBT. Respondents noted frustration that payers may prioritize the delivery of intervention components over allocating session time to build rapport. They reported that payers rarely provide reimbursement for longer (e.g., 60- to 90-min) or more frequent sessions, even though “45 min is not sufficient time” (P1226). Clinicians described that longer session time would be especially valuable for child patients and for conducting exposures in session. Clinicians indicated that they often opt to schedule clients back-to-back in order to maximize profit. They described variability in the amount of reimbursement provided by different payers, such that clinicians are less inclined to accept certain insurance providers who reimburse less for the same service. One clinician said that she only takes one insurance and “a sliding scale” (P1526). Clinicians also mentioned occasionally seeking certification in order to “re-negotiate reimbursement with insurance companies” (P8907). Lastly, clinicians indicated that high health insurance copays and deductibles are a barrier to families' treatment access.

##### Mixed methods analyses

There were no differences in descriptions of payer restrictions between clinicians who reported high vs. low organizational support; however, there were several differences between solo vs. group practice. Clinicians working in solo practice mentioned the administrative burden of dealing with insurance companies (e.g., authorization procedures, potential audits), which was not mentioned by clinicians working in group practice. Clinicians in solo practice also talked in detail about billing codes and dollar amounts from different insurance companies [e.g., “Insurance company name]'s low payment is a barrier… Even for a 90837 (billing code), it's significantly lower (than other companies)” (P1058)]. This topic was not discussed by clinicians working in group practice. Clinicians in solo practice made comments about what insurance companies want them to be doing (e.g., which billing codes to use, regulations related to frequency and duration of sessions) and more often indicated that they interpreted the billing guidelines more flexibility (e.g., billing for an in-office session even if part of the session took place in the community). Clinicians in group practice did not make comments about specific payers (e.g., which companies reimburse for what billing codes), but indicated an understanding of broad limitations of billing [e.g., “Insurance companies don't reimburse me for me to travel to (the client's) house and travel back” (P1226)].

#### Fiscal incentives

Clinicians described fiscal incentives that may incentivize EBI implementation. Clinicians had varied opinions about whether increased payment to deliver EBIs compared to other interventions would motivate them to implement EBIs; some clinicians reported that increased pay would motivate EBI use, whereas others said that they would use EBIs regardless of pay rate. Clinicians noted that payment for training would incentivize EBI use, given that cost is a barrier to attending training and that time spent in training is time spent not seeing clients. One clinician explained, “What complicates this is this kind of fee-for-service insurance-based care. Time is money. So every hour spent in a case conference or in a training session or whatever it may be is an hour less of a clinician seeing a patient” (P2621). Clinicians also mentioned that certification may in some cases incentivize training attendance. Some clinicians described that certification can provide specialized training, allow for opportunities to become a supervisor, and increase referrals to their practice, whereas others described that certification is not necessary in order to achieve specialized training, may be overly rigid and expensive, and may limit the type of referrals a provider receives. Clinicians reported that receiving CEUs is necessary but is not a primary motivator for how and why clinicians opt to attend training.

##### Mixed methods analyses

There were some differences in descriptions of fiscal incentives between clinicians working in solo vs. group practice, but no differences between clinicians with high vs. low organizational support. Clinicians working in solo practice indicated that they might be responsive to fiscal incentives (e.g., being compensated by insurance payers at a higher rate). For instance, one clinician said, “We live in a world that is monetized, so definitely increasing payment would [motivate me to use EBIs]” (P8907). In contrast, clinicians working in group practice made statements suggesting that they use EBIs regardless of fiscal incentives, such as, “If insurance would pay an extra 10 dollars per session for using motivational interviewing or something. No, I don't care about that” (P2605). Lastly, clinicians working in solo practice discussed various benefits and limitations of certification, whereas only one group practice clinician mentioned certification and said that it did not affect their practice.

### Social context

Responses related to social context included several themes, including consumer beliefs and education about EBIs, consumer demand for EBIs, and consumer reactions to EBIs presented in treatment. Clinicians noted that certain geographical locations may affect beliefs about certain interventions. For example, one clinician noted, “The whole town is crazy about internal family systems treatment” (P1058). Related to this, consumer demand for EBIs was described as variable and guided by clients' understanding about EBIs. Clinicians noted that consumers who are educated about EBIs may be more likely to request them in treatment, which may increase the likelihood that a clinician will implement them. On the other hand, many clinicians noted that families who do their own research may “come in… with some notions [about EBIs] that are accurate and some that are not” (P4610). Lastly, clinicians described that clients have mixed reactions to EBIs introduced in treatment. Some clients have positive reactions to research-based treatment “because people like to hear that things are going to work” (P6303), whereas others “might not be receptive or compliant to the treatment” (P1226) or may be “extremely resistant” (P8907) if they “just need like an open ended session and they just need to vent or they don't want the therapy to feel as formal as maybe it could be” (P1004). Clinicians described that getting client buy-in for EBIs can be difficult and noted the importance of tailoring the EBI to the individual for this reason.

Clinicians noted that consumers' understanding of and familiarity with exposure therapy varied, such that some clients “do their own research before coming in” (P4610) whereas others “don't necessarily know to come in and ask for exposure” (P3004). One clinician said, “I think the biggest problem that I encounter is that people are not compliant with it. Like people don't want to do it because it is too scary… but when we explain the rationale of these interventions to patients or clients, it makes perfect sense to them” (P1226).

#### Mixed methods analyses

There were minimal differences in perceptions of social context affecting EBI implementation for therapists in solo vs. group practice. Clinicians in group practice talked slightly more often about using psychoeducation to build client buy-in to EBIs. Differences by level of organizational support were also minimal. Clinicians with high organizational support more consistently described having clients ask them for certain types of treatment [e.g., “Some people come in and [exposure] is what they want” (P1616)]. Clinicians with low organizational support had more mixed views on whether people ask for certain types of treatment. For instance, one clinician said, “I think some adults … have done their homework and have heard that [CBT] is a recommended treatment modality for what they're coming for. But most of them are not [asking for CBT] because I see a lot of kids and teenagers” (P6303).

### Political climate

No participants made comments about the ways in which current or past political climates have influenced their use of EBIs, including exposure.

## Discussion

This study examined an understudied area of implementation research by focusing on implementation determinants in private practice mental health settings guided by the policy ecology framework. Results from this study will inform future efforts to implement EBIs in private practice settings, where a large proportion of individuals seek mental health treatment. Responses from qualitative interviews highlighted the unique considerations for this setting and potential ways to tailor implementation strategies to increase clinicians' use of EBIs, including exposure therapy. Findings demonstrate how broadening our assessment of determinants by using a policy ecology framework may also inform future implementation strategies. Specifically, findings highlight the importance of tailoring implementation strategies to address organizational, agency, and social factors specific to private practice to increase EBI implementation in this setting. Participants did not specifically identify ways in which the political context, including state or federal policies, influenced their use of EBIs; however, political-level changes would likely influence some of the themes that were described by clinicians. Mixed methods analyses identified how EBI determinants may differ for solo vs. group private practice, as well as for organizations with high vs. low organizational support. Potential implementation strategies that address these policy ecology-informed determinants are discussed below and presented in Table 2.

Given evidence that organizational culture influences EBI implementation across various healthcare settings (27), we were particularly interested in understanding how organizational context functioned as a determinant of EBI implementation in private practice settings. Studies of organizational culture and climate typically differentiate between individual and organizational levels of analysis (52). However, there are many private practice organizations that consist of only one individual. Thus, we sought to examine how individuals in such settings describe their organizational context and its influence on their EBI use. The range of responses about the level of organizational support for exposure (OISCE scores) among solo private practice clinicians (*range* = 0.00–3.06) highlights the fact that there are differences in organizational constructs even across settings that are comprised of one individual. Although it is possible that these variations reflect individual differences, they likely also reflect the larger context in which clinicians work (i.e., geographical setting, theoretical orientation of colleagues, funding structure). An example of an organizational characteristic more commonly noted by clinicians working in solo private practice organizations was that flexibility within their work setting may facilitate EBI implementation. For instance, they described having more control over their schedules and the location in which they deliver services. They also endorsed more flexibility in terms of treatment delivery, such as being able to meet clients in the community for sessions. In contrast to this, clinicians working in group private practice described having access to resources that are, by definition, not available in solo practice, such as colleagues to discuss cases with and staff who manage billing. Clinicians working in solo private practice offered ideas for implementation strategies that might address some of the barriers specific to this setting, such as building peer networks and forming external peer consultation groups to supplement the lack of colleague support. Future studies using social network analysis of clinicians in private practice may provide insights into how this is currently happening or ways in which existing networks could be enhanced to support peer consultation groups across providers (53).

For clinicians in both solo and group practice settings, there was variability in reports of organizational support for exposure implementation, which likely influences clinicians' perceptions of and use of EBIs (54). Overall, quantitative scores on the measure of organizational support for exposure (OISCE) in this sample were consistent with community settings (*M* = 1.21, *SD* = 0.86) and lower than anxiety specialty clinics [*M* = 3.62; *SD* = 0.34; (52)]. These relatively low scores suggest that existing organizational supports may be inadequate to support exposure implementation for many private practice clinicians. Consistent with the organizational factors expected to support exposure implementation (23), clinicians in practices with high organizational support described having more access to EBI training, as well as supervisory and colleague support for discussing cases. Improving access to training is likely to address multiple barriers, including: (1) increasing knowledge and (2) addressing misconceptions/beliefs about exposures that may interfere with their use (22). Group practices may benefit from ensuring that opportunities for training incorporate supervisors, consistent with recommendations from previous research [e.g., (55)]. This is likely to be more challenging for solo practices in which supervisors are unlikely to be present. As noted above, an alternative might be to create peer networks of expertise to connect clinicians across solo practices who aim to deliver EBIs. Evidence suggests that the presence of colleague or supervisor support for implementing EBIs may increase EBI use, even for clinicians who do not directly participate in EBI implementation initiatives (37). This further highlights the importance of fostering organizational cultures that support EBIs, which may look different for private practice compared to publicly-funded mental health settings.

Notably, even organizations that were described as supporting EBI use conceptually did not provide all of the necessary resources for clinicians to deliver them. Specifically, clinicians described organizational policies that restricted their ability to hold longer sessions or meet clients in the community to conduct exposures. Thus, organizations that are interested in implementing and sustaining EBI use may benefit from a regular review of organizational policies to ensure that they align with expectations that EBIs are prioritized. However, many organizational determinants stem from barriers that were identified at the agency or regulatory level, as discussed in more detail below. Even if organizations adopt policies that support EBI implementation, such as allowing clinicians to leave the office for therapy sessions or to travel with clients in the community, payer restrictions may interfere. Clinicians highlighted how factors such as the inability to bill insurance companies for longer sessions or for sessions conducted outside of the office can make it particularly challenging to implement EBIs, a common concern identified in previous research on implementation of exposure therapy (24). Creation of billing codes that permit travel and longer session lengths would likely address these barriers. This may be particularly warranted given growing evidence supporting home-based delivery of EBIs such as exposure (56–58). Providing increased education to care managers about EBIs may also facilitate the creation of such billing codes.

In this vein, responses related to the agency-context (i.e., payer restrictions and fiscal incentives) indicated that clinicians working in private practice may be disincentivized from engaging in activities for which they are not reimbursed. Clinicians described having difficulty attending training in EBIs, providing collateral contact, and conducting long enough sessions to meet clients' needs. Clinicians working in solo private practice also described being disincentivized from accepting insurance given variable or poor rates of reimbursement and the administrative burden of doing so (59). These findings are largely consistent with previous research examining EBI implementation determinants in other routine mental health care settings. For instance, Okamura et al. (60) found that EBI delivery in public sector mental health settings can incur significant costs to therapists and organizations, which may serve to disincentivize implementation. The same is likely true of EBI use in private practice settings, especially given that private practice organizations do not receive public sector financial support. Costs associated with EBI implementation were also found to vary by intervention (61), which is consistent with our finding that exposure therapy has unique challenges to its implementation. Overall, respondents in this study indicated a strong desire for compensation for services they are already providing (e.g., collateral contact), but varied in their reports of whether compensation could motivate them to change their current practice and incorporate new EBIs.

Efforts to reduce discrepancies in reimbursement across insurance providers and compensate clinicians for training and for clinical services they are already providing (e.g., collateral contact) is one potential method to increase EBI use. Previous research examining stakeholder preferences related to implementation strategies has demonstrated that stakeholders across several groups (i.e., clinicians, supervisors, agency executives, payers) agree that financial incentives are the most useful category of implementation strategies (61, 62). In terms of specific financial incentives, payers would prefer to provide compensation for EBI delivery rather than preparation time (61). Providing an enhanced reimbursement rate for EBI use compared to other interventions may serve to encourage EBI implementation for some clinicians. However, in past research and in the current study, clinicians also identified a strong desire to be compensated for EBI preparation time and training, which has been rated as a less preferred option by payers (61). A potential lower-cost alternative to compensation for preparation time that might facilitate EBI implementation would be to allot funding for therapy supplies. This might be a more feasible incentive that payers could offer to support clinicians in implementing EBIs. Alternatively, the creation and dissemination of freely available and easily accessible (e.g., online) toolkits that incorporate active components, such as reminders, have been shown to support the implementation of EBIs [e.g., (63)]. Furthermore, advocates of “digital apothecaries,” or online repositories for digital interventions (e.g., websites, mobile apps, digital tools) suggest that such resources may be particularly helpful for supporting private practice clinicians and their clients in using EBIs (64). Future implementation research should examine whether tailored online toolkits and digital apothecaries reduce barriers to EBI use and defray costs related to supplies. It is also worth noting that many clinicians mentioned that physical supplies are necessary when working with children (e.g., toys, games, prizes). In a group practice, it might be feasible for clinicians to pool funds for shared supplies. However, in solo practice this may be more challenging. Future implementation research should examine whether stakeholder preferences for financial incentives lead to meaningful behavior change, and whether lower-cost interventions such as providing supplies are effective at changing behavior or maintaining use of EBIs in private practice settings.

In addition to organizational- and agency-level factors, social context was also described as a determinant of EBI implementation. Specifically, clinicians reported that they may be more likely to implement EBIs if patients request them or respond positively to their introduction in treatment. This is consistent with prior research indicating that concerns about patient and caregiver reactions to an EBI (exposure therapy) are a common barrier to implementation (22). Consumer demand for and reaction to EBIs may be influenced by consumer education, geographical location, and culture. Respondents suggested that some patients may respond more negatively to exposure therapy compared to other EBIs. These findings are consistent with existing literature suggesting the need for additional efforts to increase consumer education about and demand for EBIs, including exposure therapy. Direct-to-consumer marketing strategies (42) aimed at increasing consumer demand for EBIs may incentivize more clinicians to use EBIs. Importantly, such marketing strategies may be most effective if tailored to specific subgroups of consumers (65). Thus, continued efforts are needed to examine how such direct-to-consumer marketing strategies should be tailored to consumers receiving services in public vs. private practice mental health settings.

Although clinicians were asked about determinants at the political level of the policy ecology, there were no themes that emerged in this category. One possibility is that considerations related to legislation were perceived by clinicians as too distal to have a direct influence on their behavior. Responses tended to focus on more proximal determinants of clinicians' use of EBIs in their current setting, such as the types of referrals they receive and the geographical location in which they work. While some participants mentioned whether their graduate training emphasized EBIs, they did not describe how this might be linked to political or administrative issues, such as accrediting requirements. Another possible explanation for the lack of findings in this domain is that private practice clinicians perceived the political context as being less relevant to their practice given their decision to operate in private practice settings; they may have selected this setting for its decreased regulatory requirements compared to publicly-funded mental health settings. More focused inquiry into how specific political issues, such pre-service training, loan forgiveness programs, and mental health parity might affect private practice clinicians should be the focus of future research.

The recommendations generated from our findings are a first step toward increasing EBI use in private practice settings. A key strength of this study is the application of an existing implementation framework (i.e., policy ecology framework) to a novel context (i.e., private practice mental health). Given the proliferation of implementation frameworks, applying existing frameworks to new contexts has been identified as a priority for implementation research (66). Additional methodological strengths of this study include the use of purposive sampling to recruit clinicians with and without training in a difficult-to-implement EBI, exposure therapy. Similarly, the inclusion of clinicians working in different types of private practice settings (i.e., solo vs. group private practice) provides insight into EBI implementation in a range of settings in which clinicians might work.

Results should also be interpreted in the context of limitations. The small sample size and lack of ethnic and racial diversity of participants included in this study may limit the generalizability of the study's results. In particular, there are likely to be barriers faced by clinicians of color and their patients that may not be reflected in findings, such as the role of racial discrimination and bias, the need for resources in other languages, and other systemic considerations not represented in these results. Participants included in this study were clinicians located in New England, where clinical practice may differ from other geographical regions. For example, clinicians working in private practice in New England may accept public insurance, which differs from other regions of the United States. Additionally, all clinicians included were those who were willing to participate in a research study, and many had previously participated in a clinician training research study conducted by the same organization. Willingness to participate in a research study may suggest that these clinicians are open to research more broadly and may hold more positive attitudes toward EBIs than clinicians who are unwilling to participate in a research study. In addition, this study did not measure actual EBI or exposure use in clinicians' routine clinical practice. Future research should quantitatively assess whether the identified implementation determinants influence clinicians' EBI use. Finally, limited information was collected on the exact nature of previous training in exposure therapy, and clinicians likely varied widely in the amount of training they have previously received in EBIs.

Despite its limitations, this study provides novel information about the multi-level factors that influence the implementation of EBIs in private practice and supports the use of a policy ecology framework to inform the generation of setting-specific implementation strategies. Respondents in this study cited various organizational, agency-related, and social barriers to the implementation of EBIs in routine clinical practice, which informed suggestions for implementation strategies that may address these barriers. Future research should examine the feasibility, acceptability, and effectiveness of the suggested implementation strategies to increase EBI use in private practice. Future research should also examine the cumulative effects of multiple implementation strategies to target different ecological levels and maximize the likelihood of EBI implementation.

## Data availability statement

The datasets presented in this article are not readily available due to the sensitive and potentially identifiable nature of interview transcript data. Requests to access the datasets should be directed to HF, hannah_frank@brown.edu.

## Ethics statement

The studies involving human participants were reviewed and approved by Lifespan Institutional Review Board. Written informed consent for participation was not required for this study in accordance with the national legislation and the institutional requirements.

## Author contributions

HF conceived of and designed the research study, acquired and analyzed the data, interpreted the data, drafted the manuscript, and substantially revised it. LM analyzed and interpreted the data and drafted sections of the manuscript. JF and KB helped conceive of and design the research study and substantially revised the manuscript. All authors approved the submitted version, have agreed to be accountable for the contributions, and attest to the accuracy and integrity of the work, even aspects for which the authors were not personally involved.

## Funding

This study was provided by a grant from the National Institute of Mental Health awarded to HF (T32MH019927, PI, Spirito). The funding bodies did not have any direct role in the manuscript idea or writing of the manuscript.

## Conflict of interest

The authors declare that the research was conducted in the absence of any commercial or financial relationships that could be construed as a potential conflict of interest.

## Publisher's note

All claims expressed in this article are solely those of the authors and do not necessarily represent those of their affiliated organizations, or those of the publisher, the editors and the reviewers. Any product that may be evaluated in this article, or claim that may be made by its manufacturer, is not guaranteed or endorsed by the publisher.
