# Supplementary material for: Expanding the reach of evidence-based mental health interventions to private practice: Qualitative assessment using a policy ecology framework
Source: Front Health Serv. 2022 Jul 22;2:892294. doi: 10.3389/frhs.2022.892294 (PMC10012822; doi:10.3389/frhs.2022.892294)
Supplement: Supplementary file 1 [file Data_Sheet_1.pdf]

Qualitative Interview Guide

General Introduction Script:

Thank you for agreeing to participate. My name is [**Interviewer's Name]**, and I am affiliated with Brown University and Bradley Hospital.

As part of this study, we would like to learn more about your use of evidence-based practices in your work as a therapist in private practice. We are particularly interested in your use of exposures. *Exposures are defined as structured activities that gradually help clients face their fears. The purpose of exposures is to intentionally elicit uncomfortable feelings (i.e., anxiety) in a supported way to help clients overcome their fears. Exposures can be imaginal or take place in real life.* We know that regularly using evidence-based practices and exposures can sometimes be difficult. There are probably advantages and disadvantages to using evidence-based practices and exposures. Today I will ask about your experience with using exposures in clinical practice, as well as your needs for training and consultation in this area. I have prepared some questions that will take no more than one hour to answer. You can spend as much or as little time as you like answering each question. At the end of the interview, you will receive $50 for your participation.

Our goal is to learn more about your perspective on exposure therapy and other evidence-based practices. It's important for you to know that there are NO right or wrong answers. We are interested only in *your* opinions and perceptions.

Do you have any questions?

1. Tell me about the primary presenting problems and treatment modalities that you use in your practice.

[Probes:
- *What are the modalities that you use the most?*
- *Approximately what percent of the clients you treat have clinically significant anxiety?]*

2. When you hear the words, "evidence-based practices," what thoughts come to mind?

3. Tell me about your experience using evidence-based practices (EBPs) with your clients.

[Probes:
- *Have you ever used them? [always ask if they did not respond to the first question with an answer about their personal experience using EBPs]*
- *How do you decide when or with whom to use them? Or when not to use them?*
- *Are there elements of EBPs that you particularly like or dislike?*
- *Are there advantages or disadvantages to using EBPs?*
- *How do clients react when suggest using EBPs?*
- *How did you get your training in EBPs? Graduate school? Continuing education workshops? Conferences?*
- *What are your perceptions of using outcome assessments for EBPs?*
- *Do you ever follow a specific protocol? (If so, tell me about it).]*

4. When you hear the words "exposure therapy," what thoughts come to mind?

5. Tell me about your experiences and perceptions of using exposures with your clients. [Provide definition if needed]

[Probes:
- *Have you ever used them? [always ask if they did not respond to the first question with an answer about their personal experience using exposures]*
- *How do you decide when or with whom to use them? Or when not to use them?*
- *Are there elements of exposures that you particularly like or dislike?*
- *Are there advantages or disadvantages to using exposures?*
- *How do clients react when suggest using exposures?*
- *How did you get your training in exposures? Graduate school? Continuing education workshops? Conferences?]*

6. What are some factors that make it hard to use EBPs/exposures?

[Probes:

- *Are there any client characteristics that make it particularly hard?*
- *Anything about your organization that makes it hard? Are you able to leave your clinic/office?*
- *Anything about your supervisor?*
- *Anything about the amount of time you have to prepare?*
- *Anything about insurance companies/payers? Does it differ for private/public insurance?*
- *Any state or federal policies or public perceptions?*
- *Is there anything that makes it hard for you personally/emotionally?*

- *Is there anything related to the procedures of completing them that makes it difficult to use them?]*

7. What are some factors that make it easier to use EBPs/exposures?

[Probes:

- *Are there any client characteristics that make it easier?*
- *Anything about your organization?*
- *Anything about your supervisor?*
- *Anything about insurance companies/payers? Does it differ for private/public insurance?*
- *Any state or federal policies or public perceptions?*
- *Is there anything that makes it easier for you personally/emotionally?*
- *Is there anything related to the procedures of completing them that makes it easier to use them?]*

8. What kinds of training and consultation would be helpful to support you in using EBPs/exposure therapy?

[Probes:

- *Tell me about what kinds of training/consultation opportunities you have received in the past and how you have learned about them. (If in peer consultation, tell me about the group, how it formed, how frequent, etc.).*
- *What kind of training would be feasible/acceptable?*
- *What kind of training/consultation would be ideal in terms of time, frequency, and modality (e.g., online, in-person, individual, group)?*
- *Would kind of incentives would motivate you to engage in training/consultation? CEs? Additional payment for training or for sessions using EBPs? Paid time off for training? Any other incentives?*
- *On what topics are you most interested in receiving training/consultation?]*

9. Tell me about what does or would motivate you to use EBPs/exposure therapy?

[Probes:

- *What incentives do you currently receive (if any) to use EBPs/exposure therapy?*
- *What would make you more or less likely to use them?*
- *What could payers do to support you in using EBPs/exposure therapy?*
- *What kinds of policies would support your use of EBPs/exposure therapy?*
- *Do you receive or would you want to receive an enhanced rate for using EBPs? What about CEs for attending training/consultation? Being labeled as a provider with a particular type of training? Being considered a preferred provider with insurance companies if you use certain EBPs? Increased referrals? Public recognition?*

10. If you were the head of a large private practice with unlimited funds and unlimited power, what would you do to improve clinician training in EBPs and exposure therapy? To increase the use of EBPs/exposure therapy within your practice? To support long-term use of EBPs/exposure therapy?
